# Recent Advances in Understanding the Roles of Pectin as an Active Participant in Plant Signaling Networks

**DOI:** 10.3390/plants10081712

**Published:** 2021-08-19

**Authors:** Yesol Shin, Andrea Chane, Minjung Jung, Yuree Lee

**Affiliations:** 1 School of Biological Sciences, Seoul National University, Seoul 08826, Korea; yshin333@snu.ac.kr (Y.S.); chane.andrea@gmail.com (A.C.); mj.jung@snu.ac.kr (M.J.); 2 Research Center for Plant Plasticity, Seoul National University, Seoul 08826, Korea; 3 Plant Genomics and Breeding Institute, Seoul National University, Seoul 08826, Korea

**Keywords:** pectin, cell wall remodeling, outside-in signaling

## Abstract

Pectin is an abundant cell wall polysaccharide with essential roles in various biological processes. The structural diversity of pectins, along with the numerous combinations of the enzymes responsible for pectin biosynthesis and modification, plays key roles in ensuring the specificity and plasticity of cell wall remodeling in different cell types and under different environmental conditions. This review focuses on recent progress in understanding various aspects of pectin, from its biosynthetic and modification processes to its biological roles in different cell types. In particular, we describe recent findings that cell wall modifications serve not only as final outputs of internally determined pathways, but also as key components of intercellular communication, with pectin as a major contributor to this process. The comprehensive view of the diverse roles of pectin presented here provides an important basis for understanding how cell wall-enclosed plant cells develop, differentiate, and interact.

## 1. Introduction

The cell wall is a cellulosic extracellular matrix that surrounds plant cells. This material not only provides structural support for cells and tissues, but also plays essential roles during such processes as cellular proliferation, differentiation, and defense responses [1,2]. The word “wall” conjures an image of something solid and static, but the cell wall of plants, especially the primary cell wall, is very dynamic and can take on cell type-specific properties and adapt to various environmental changes. The diverse structure of pectin is a major contributor to this cell wall plasticity.

**Figure 1:**
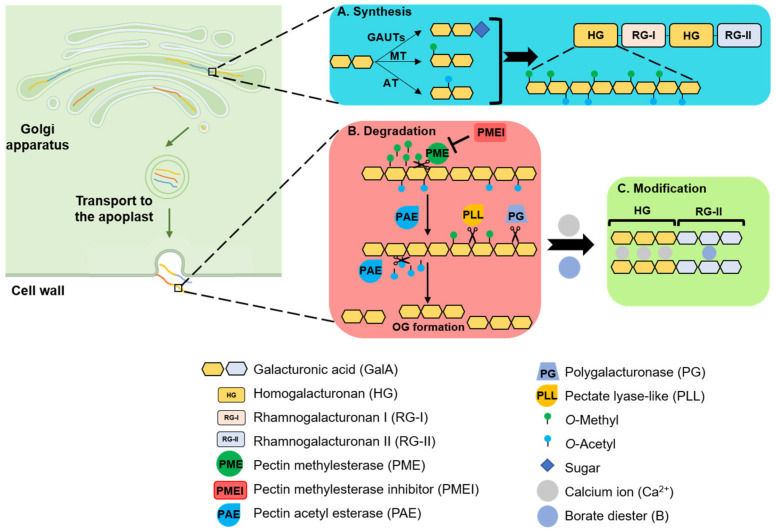
An overview of the synthesis, degradation, and modification of pectin in the plant cell wall. (**A**) Pectins—structurally complex polysaccharides including homogalacturonan (HG), rhamnogalacturonan I (RG-I), and rhamnogalacturonan II (RG-II)—are synthesized in the Golgi apparatus. HG is synthesized by the glucuronosyltransferase (GAUT) family of enzymes and modified by methyltransferase (MT) and acetyltransferase (AT). (**B**) The highly methylesterified pectin is delivered to the cell wall surface via vesicle-mediated transport and incorporated into the cell wall. HG is selectively demethylated via the wall-bound pectin methylesterases (PMEs) and deacetylated via pectin acetylesterases (PAEs). PME activity is controlled by the proteinaceous pectin methylesterase inhibitor (PMEI). Pectinolytic enzymes such as polygalacturonase (PG) and pectate lyase-like (PLL) are important for HG depolymerization, which results in the formation of oligogalacturonides (OGs). (**C**) Antiparallel polyuronate chains of HG form egg-box dimers with Ca^2+^, and HG backbones of the RG-II polysaccharides are covalently linked via borate diester crosslinks.

Pectin, along with cellulose and hemicellulose, is a major component of the primary cell wall, accounting for up to 35% of primary cell walls in dicots and non-graminaceous monocots [3]. Due to its abundance in the middle lamellae, pectin was initially considered to function primarily in intercellular adhesion [4,5]. However, pectin is involved in a variety of cellular processes, including cell fate specification, morphogenesis, intercellular communication, and environmental sensing [6,7,8,9]. The pectin matrix is composed of heterogeneous polymers with diverse pectic polysaccharides, primarily homogalacturonan (HG), the substituted galacturonan rhamnogalacturonan (RG-II), and rhamnogalacturonan I (RG-I) [10]. Pectin is synthesized by various interconversion enzymes, including glycosyltransferase (GT), methyltransferase (MT), and acetyltransferase (AT). During pectin biosynthesis, sugars are transferred from an activated donor to an acceptor, leading to the elongation of the sugar polymer [10,11]. Newly synthesized pectin from the Golgi apparatus is secreted into the cell wall, where it undergoes various modifications such as demethylesterification and deacetylation that affect the mechanical properties of the cell wall [12,13] (Figure 1).

Although our understanding of pectin biosynthesis and modification is still limited, this review addresses significant recent advances, including the identification of the enzymes involved in these processes. In addition, we describe insights into pectin’s involvement in various dynamic cellular mechanisms that regulate plant development. We explore, in depth, the emerging view that pectin is not a passive outcome but an active participant in signaling processes during plant development and interactions with the environment.

## 2. Biosynthesis and Modification of Pectin

### 2.1. Biosynthesis of Pectin

Pectins—structurally complex polysaccharides including HG, RG-I, and RG-II—are synthesized in the Golgi apparatus and secreted into the cell wall [10,14]. HG, which is composed of approximately 65% pectin, is synthesized by the well-characterized glucuronosyltransferase (GAUT) family of enzymes [15,16]. GAUT transfers galacturonic acid (GalA) from uridine-diphosphate-GalA (UDP-GalA) onto HG acceptors [17]. Following the identification of the first *GAUT* gene, *GAUT1*, 14 additional members of the GAUT family and 10 members of the GAUT-like family were identified [10]. Although their exact roles remain unclear, there is growing evidence that GAUT family members have diverse functions in HG synthesis. GAUT1 and GAUT7 are type II membrane proteins with a single N-terminal transmembrane-spanning domain and are both localized to the Golgi. The N-terminal region of GAUT1, including the transmembrane domain, is cleaved in vivo, raising the question of how solubilized GAUT1 is retained in the Golgi, the site of HG biosynthesis. GAUT1 and GAUT7 form a complex, and the retention of GAUT1 in the Golgi is dependent on GAUT7, suggesting a non-catalytic GAUT1-anchoring role for GAUT7 [16]. HG synthesis via GAUT heterocomplexes was recently reported in pollen tubes: GAUT5, GAUT6, and GAUT7 are redundantly required to localize GAUT1 to the Golgi and for HG biosynthesis and male gametophyte development [15]. The *gaut5* and *gaut7* single mutants, the double mutants (*gaut5 gaut6*, *gaut5 gaut7*, and *gaut6 gaut7*), and the heterozygous triple mutant (*gaut5 gaut6 gaut7*^+/−^) show increasingly severe defects in pollen tube elongation, resulting in male infertility in the heterozygous triple mutant [15]. The specialization of different GAUT family members for scaffolding and/or catalytic functions may contribute to the ability of plant cells to generate complex and variable cell walls. In addition, different GAUT enzymes can synthesize functionally distinct HG domains, providing a new perspective on pectins as a glycan family that exists as distinct homoglycans, heteroglycans and proteoglycans [18,19]. The elucidation of the unique molecular functions of each member of the GAUT and GAUT-like families, and the identification of proteins associated with various GAUT1-containing complexes, would provide a better understanding of the mechanisms enabling cell type- and developmental stage-specific HG synthesis.

RG-II is a pectic polysaccharide of high structural complexity, consisting of an HG backbone with four distinct side chains (A–D) composed of 12 different monosaccharides [20]. RG-II exists predominantly as a dimer in which the HG backbones of the RG-II polysaccharides are covalently linked via borate diester crosslinks, yielding a macromolecular pectin network [21,22]. In vitro experiments showed that RG-II dimerization at pH 3–4 is faster in the presence of divalent cations such Sr^2+^, Ba^2+^, and Pb^2+^ [20,23]. Although neither a specific enzyme that catalyzes the borate crosslinking of RG-II nor a putative GT for RG-II biosynthesis has been identified, the biological importance of maintaining the structural integrity of RG-II was demonstrated [24]. In *Arabidopsis thaliana*, *MURUS1* (*MUR1*) encodes GDP-_D_-mannose-4,6-dehydratase, which synthesizes the _L_-Fuc found in RG-II. The *mur1* mutant exhibits a fragile cell wall, along with changed rosette leaf morphology and dwarfism. Treatment with boric acid rescues the leaf expansion defect of *mur1*, suggesting that RG-II crosslinking is essential for normal leaf expansion and plant growth [25,26]. Following the MUR1 study, several major monosaccharide synthetases and borate crosslinking enzymes for the RG-II side chain were identified [24].

RG-I, a branched pectic polysaccharide consisting of [2)-a-L-Rha(1-4)-a-D-GalUA(1-] disaccharide repeats, constitutes approximately 25% of the pectin molecules found in terrestrial plants [27]. Our understanding of the main chain biosynthesis of RG-I is limited, and rhamnosyltransferase (RRT), the enzyme responsible for the biosynthesis of the RG-I backbone, was only recently identified in eudicot plants [28,29]. Arabidopsis contains four RRTs: RRT1–RRT4. Recent studies revealed a role for RRT1 in the formation of seed coat mucilage [28]. *RRT1* is highly expressed in siliques containing developing seeds, and a loss-of-function mutation in *RRT1* reduces RG-I levels in the seed coat mucilage as well as the volume of mucilage. Three other RRT proteins exhibit different tissue-specific expression patterns in Arabidopsis: RRT2 in leaves and flowers, RRT3 in dry seeds, and RRT4 in pollen tubes [28]. These specific expression patterns suggest that each RRT is primarily responsible for RG-I synthesis in the corresponding organs, but functional analysis is needed to confirm this possibility.

### 2.2. Modification of Pectin (Methylation and Acetylation)

Newly synthesized HG is methylated in the Golgi apparatus before being secreted into the cell wall [14]. MT activity is detected in a Golgi-enriched fraction, and several putative MT enzymes were identified in Arabidopsis, including QUASIMODO2 (QUA2), QUA3, COTTON GOLGI RELATED2 (CGR2), and CGR3 [14,30,31,32]. QUA2 has MT activity in vitro [33]. QUA2, also known as TUMOROUS SHOOT DEVELOPMENT2 (TSD2), is one of 29 QUA2-related type II membrane proteins and contains a putative MT domain [32]. In *qua2* mutants, HG contents are reduced by 50% and cell adhesion is severely affected in hypocotyls and cotyledons. *qua2* mutants also show defects in cellulose biosynthesis and microtubule stability, suggesting that pectin plays a role beyond acting as a simple “glue” in plant cells [33].

After the highly methylesterified pectin is delivered to the cell wall surface via vesicle-mediated transport and incorporated into the cell wall, HG is selectively demethylated via the wall-bound pectin methylesterases (PMEs). The distribution of methyl groups on the HG backbone can affect cell wall stiffness, as shown using atomic force microscopy to analyze the mechanical properties of the cell wall [34]. Calcium-dependent gelation is one of the most important properties of pectin, and the classic egg-box model is widely accepted to explain the correlation between the degree of methylesterification of HG and cell wall stiffness [13]. According to the model, antiparallel polyuronate chains of HG form egg-box dimers with Ca^2+^ and further aggregate laterally to form multimers. PME can remove methyl groups on HG to produce consecutive free carboxyl groups with which the Ca^2+^ interacts to create a pectic gel [35]. On the other hand, PME sometimes has the opposite effect: low methylesterification of HG by PME provides a cleavage site for pectin-degrading enzymes such as polygalacturonase (PG) to induce depolymerization, and the protons released by the action of acidic PME promote the activity of cell wall-degrading enzymes, thereby promoting cell wall loosening. Therefore, it is very important to precisely control PME activity. Proteinaceous inhibitors known as pectin methylesterase inhibitors (PMEIs) bind to PME and regulate its activity [35].

In addition to methylesterification at the C-6 carboxyl positions, galacturonyl residues in the backbones of pectic polysaccharides are often *O*-acetylated at the C-2 or C-3 hydroxyl positions. These modifications affect the physical and rheological properties of the polysaccharide, including the gelation solubility and hydrophobicity of the polymer [12,36,37]. Several lines of evidence suggest that the *O*-acetylation of wall polysaccharides occurs during their synthesis in the Golgi lumen and that these polysaccharides can be further modified by apoplastic *O*-acetylesterases following their deposition on the cell wall [38].

The degree of *O*-acetylation of pectin changes during the growth and differentiation of plant tissues and in response to environmental conditions [39] and involves at least two types of enzymes: ATs and acetylesterases [36]. ATs trans-acylate the sugar residues of polymers [40], and acetylesterases cleave the ester bond between a glycosyl carbon and an acetyl group, enabling the release of the acetate from the polysaccharide [41]. Although the phenotypes are purely descriptive and the causal relationship is uncertain, several reports indicate that changes in pectin *O*-acetylation affect a variety of processes, including photomorphogenesis and defense responses [42,43]. POWDERY MILDEW RESISTANT5 (PMR5) in Arabidopsis is a functional AT that mediates the acetylation of GalA in pectin [44]. PMR5 belongs to the trichome birefringence-like (TBL/DUF231) family, which contains other members involved in cell wall acetylation and possesses an esterase domain that is evolutionarily conserved with the fungal AT *C. neoformans* Cas1p protein. The *pmr5* mutant was initially identified based on its resistance to powdery mildew via an ethyl methanesulfonate (EMS)-generated forward genetics screen [45]. Since the powdery mildew resistance in *pmr5* is independent of the salicylic acid, jasmonic acid, and ethylene defense pathways, the underlying mechanism had remained a mystery. Recent findings not only link *pmr5*-mediated resistance to cell wall structure, but also point to the importance of the acetylester status of pectin polysaccharides.

### 2.3. Degradation of Pectin

Pectin is degraded via pectinolytic enzymes, including pectin esterase, PGs, pectate lyases (PLs), and pectin lyases (PNLs) [46,47]. Pectin esterase specifically removes the methoxy and acetyl residues of polygalacturonic acids, PGs degrade pectin via hydrolysis of the glycosidic linkages, and PLs and PNLs are responsible for the eliminative cleavages [48].

PGs cleave polygalacturonans via hydrolysis of the glycosidic linkages between GalA residues [48]. The PG gene family in Arabidopsis is large, containing 72 genes. Although only a few PG genes have been characterized, PGs contribute to a wide range of cellular processes, including cell proliferation, cell expansion, morphogenesis, and cell separation [49,50,51,52,53,54]. A mutation in *QUARTET3*, encoding an enzyme with PG activity, prevents microspore separation during pollen development [49]. In addition, mutation of AT2G41850, a PG gene that is specifically upregulated during floral organ abscission, delays abscission [55,56].

PLs and PNLs perform the eliminative cleavage of α-1,4-d galacturonic linkages, producing a Δ-4,5 unsaturated oligosaccharide at the non-reducing end of the product. PNLs require the methylesterification of GalA, whereas PLs are more specific for non-methylesterified or slightly methylesterified HG. Until recently, plant pathogens were thought to be the primary source of PLs and PNLs, the action of which makes plant tissues brittle. However, PL-like (PLL) sequences are abundant in the plant genome, suggesting that these enzymes play important roles in normal plant processes. Of the PNLs and PLs, only PLs were found in plants and characterized biochemically [57]. In Arabidopsis, for example, 26 genes encode PLL proteins [58]. PGs were thought to be the main enzymes responsible for fruit softening. However, pectin solubilization and degradation occur even when PG activity is very low or absent [59,60], highlighting the importance of PLLs in this process. PLLs are also involved in a variety of biological processes, such as the generation of elicitor-active oligogalacturonides [61], fruit ripening [62,63], and xylem development [64]. Although further analysis is needed to elucidate the precise roles of PLLs and PGs, these findings suggest that the balance between the synthesis, modification, and degradation of pectin allows for the heterogeneous deposition of pectin to help ensure the proper biological functions of the cell.

### 2.4. Covalent Linkages between Pectin and Xyloglucan

Two independent polymer networks, a cellulose–xyloglucan network linked by hydrogen bonds and a pectic network linked by Ca^2+^ bridges, were proposed in widely accepted cell wall models. However, recent and growing evidence suggests that there is communication between the two networks and that pectin-linked xyloglucans may play a tethering role in primary cell wall architecture [65,66,67]. The xyloglucan-pectin (probably RG-I) linkage was detected in a wide variety of angiosperm cell-suspension cultures [67]. The presence of this xyloglucan–pectin linkage in most angiosperms, despite extensive changes in xyloglucan structure and differences in cell wall composition, suggests that this structure is evolutionarily conserved and may be required for effective cell wall structure and function. Enzymes that form xyloglucan–pectin bonds were also identified [68]. The molecular mass and composition of xyloglucans can be altered following their deposition into plant cell walls through the activities of xyloglucan endotransglycosylases (XETs) and xyloglucan endohydrolases (XEHs), forming the xyloglucan endotransglucosylase/hydrolase (XTH) group of enzymes [69]. In barley, HvXET3 and HvXET4 have hetero-transglycosylation activity as well as homo-transglycosylation activity [68]. They use xyloglucans as donors and pectin fragments as acceptors to form a xyloglucan–pectin bond. Unlike cell-suspension cultures, in which up to 50% of the xyloglucan in the cell wall is covalently bound to pectin [70], the amount of xyloglucan–pectin complex is very small in plants [71,72]. Therefore, further validation of its effect on wall integrity or wall extensibility in plants is required. Studies of mutants lacking this enzymatic activity could verify the significance of the xyloglucan–pectin linkage on the cell wall structure.

## 3. The Roles of Pectin in Stem Cell Maintenance

Stem cell differentiation has a direct impact on plant architecture. Many transcriptional and hormonal signals that regulate meristem activity were identified [73,74,75,76,77,78,79,80,81]. Little is known about whether the plant cell wall plays a role corresponding to that of the extracellular matrix (ECM) of animals in stem cell maintenance. Reciprocal stem cell-ECM interactions in animals are being actively studied, and the effects of the ECM on cell growth, viability, cell migration, and cell fate, as well as its role as a structural scaffold, are being elucidated [82,83]. The functional similarity between the animal ECM and plant cell wall raises questions about the possible influence of the cell wall on stem cell maintenance in plants.

Early studies of embryogenesis in the alga *Fucus spiralis* provided clues that the cell wall might play an essential role as a cell fate determinant [84]. *F. spiralis* protoplasts removed from their cell walls are dedifferentiated, but their fates become limited again when they are placed inside new cell walls. When an isolated protoplast contacts the cell wall of another cell type, the fate of the protoplast changes, suggesting that the cell wall plays a role in maintaining the differentiated state of a cell and directing cell fate during plant development. Further support for this idea comes from a study of the multifunctional roles of the zygotic cell wall in early embryogenesis in tobacco, which revealed that the original zygotic cell wall is essential for maintaining the apical-basal polarity and proper orientation of cell divisions of the embryo in vitro [85]. Although the molecular mechanisms underlying these findings are largely unknown, these results raise the possibility that the cell wall is actively involved in stem cell maintenance.

How might the cell wall influence meristem activity? Recent studies explored the possibility that changes in cell wall elasticity driven by pectin modifications are involved [86,87]. De-esterification of pectin occurs during flower primordia formation in subepidermal tissue layers of Arabidopsis, which contributes to an increase in elasticity of these layers, leading to extemporal organogenesis [88]. Perturbation of the esterification status of pectin by overexpression of *PMEI3* or *PME5* dramatically alters the phyllotactic pattern of inflorescences [86,88]. BELLRINGER (BLR), a transcription factor that negatively regulates the expression of *PME5* in Arabidopsis meristem, was also identified [89]. Mutation of the *BLR* gene leads to ectopic expression of *PME5* in meristem and alters phyllotaxis. These results suggest that pectin de-esterification is necessary and sufficient to trigger the initiation of organs in the inflorescence meristem.

The esterification state of pectin also affects rhyzotaxis: esterified and de-esterified pectin are differentially localized around emerging lateral root primordia (LRPs). Perturbation of the pectin esterification state through the overexpression of *PME5* or *PMEI3* leads to a loss of lateral root (LR) formation [9]. Pectin appears to have an effect beginning at the initial stages of LR formation. The spacing of LRs along the primary axis of the root is established when selected pericycle cells become LR founder cells. Although the molecular mechanism underlying the selection procedure remains largely unknown, oscillating gene expression mediates LR positioning by forming prebranch sites, where LRPs subsequently emerge [90,91,92]. Altering the pectin esterification status affects the expression of *CLE44::GFP* (which marks the nascent LRPs) and *DR5::LUC* (which marks the prebranch sites). These findings suggest that the balance between pectin esterification and de-esterification is critical for the functioning of the root clock [9]. In addition, overexpressing *PMEI5* affects the orientation of cell division, cell size, and cell number in the root meristem zone [93]. Taken together, these findings indicate that the esterification status of extracellular pectin affects the properties of meristem cells, though how this occurs remains a mystery.

## 4. The Roles of Pectin in Cell Elongation and Morphogenesis

### 4.1. Turgor Pressure and Cell Wall Deformation

Plant cell differentiation most often leads to differential growth to form a specific cell shape, which involves dramatic changes in the cell wall. This process is accompanied by an increase in the size of the central vacuole. The prevailing view is that the vacuole, turgor pressure, and the cell wall are essential factors in determining cell elongation and cell shape [94] and that an increase in turgor pressure caused by the increase in vacuole size induces cell wall deformation [95]. However, growing evidence suggests that rather than functioning as a static structure, the cell wall is an active participant in this process and that mutual interactions among these factors are important for regulating cell shape.

The central vacuole is the largest organelle in mature plant cells and a key factor in determining cell size [96,97,98]. Restricting the size of the vacuole inhibits cell elongation [98], and increasing the size of the vacuole enables rapid cell elongation with relatively little de novo cytoplasmic production [99]. In addition, cell wall acidification and subsequent loosening are required for cell expansion [100]. Despite this evidence for the positive roles of the vacuole in cell elongation, little is known about the mechanisms that explain the direct relationship between the two. A recent study investigated whether vacuoles drive cell elongation and uncovered a mechanism that detects and responds to changes in the cell wall, leading to intracellular expansion of the vacuole [99]. Changing the properties of pectin via treatment with epigallocatechin gallate (EGCG), a natural inhibitor of PME, not only inhibits cell elongation, but also limits the intracellular expansion of the vacuole. Cell wall changes are sensed through the interaction of extracellular LEUCINE-RICH REPEAT EXTENSIN (LRX) with the receptor-like kinase FERONIA (FER), transducing these signals into the cell [99]. This finding, together with the observation that cell expansion induced by auxin-mediated cell wall acidification also requires the action of FER [101], suggests that cell wall loosening induced by cell wall acidification, rather than vacuole expansion, drives cell expansion and that the status of pectin is vital in the process.

The difficulty of observing the status of pectin in living cells of plants is a technical limitation, which single-cell models can overcome. Recently, the unicellular alga *Penium margaritaceum* was introduced as a single-cell model for studying plant cell wall structure and developmental dynamics [102,103]. Its genome sequence was released [104], and it was found that *Agrobacterium*-based transformation is possible [105]. One advantage of using *P. margaritaceum* is that it allows immunofluorescence-based labeling in living cells. Monoclonal antibodies, which are currently available for higher plant cell wall studies, can be applied to visualize the precise location of various polymers in the wall in vivo, which, combined with endomembrane reporters and various mutants, will be a powerful tool to study development-dependent cell wall dynamics.

### 4.2. Cell Wall Mechanics and Epidermal Patterning

Because the cell wall imposes major biophysical constraints on plant cell growth and morphogenesis, it is important to understand how the wall structure and molecular interactions of polysaccharides define their biomechanical properties. Cell wall *loosening* refers to a molecular process that results in a relaxation of wall stress that allows water absorption, which is irreversible and essential for cell growth [106]. By contrast, cell wall *softening* refers to a process that makes the cell wall more susceptible to deformation by mechanical forces, which does not mean that the wall can grow faster [106]. How different aspects of cell wall dynamics are defined by different wall components is poorly understood, in part due to the technical limitations of complex cellular systems. Studies with a cell-free system using the onion epidermal wall in combination with various wall lytic enzymes show that loosening, softening and other cell wall properties are not tightly coupled: tensile mechanics are highly dependent on the cellulose network, whereas indentation mechanics are sensitive to pectin [107]. In addition, PME treatment induces changes in nanoindentation but does not affect tensile elasticity [108]. These results indicate that the biomechanical response to enzymatic action has selectivity, which is different from what is expected in general models of cell wall structure. Based on the observations of onion skin epidermis, a computational model was developed to simulate wall-stretching experiments and uncover the physical basis for wall plasticity [109]. Although this model may differ in detail from the actual cell wall in that it is based on the results of cell-free systems and excludes the action of expansins and other wall-modifying proteins, it provides insight into how the physical interactions between wall polysaccharides are collectively implemented into complex tensile behaviors.

The jigsaw patterns of Arabidopsis leaf epidermal cells (pavement cells) are used as models for understanding the complex morphogenetic mechanisms of plants [110]. While early research mainly focused on identifying internal factors involved in this process, such as auxin, AUXIN-BINDING PROTEIN1 (ABP1), Rho-of-plant (ROP) small GTPases, and the cytoskeleton [111,112], recent research includes external factors such as the cell wall and mechanochemical properties [8,113,114,115]. The spatiotemporal dynamics of the cell wall have been investigated using genetic and mechanical imaging methods, and various models have been proposed to explain the formation of the undulations in cell shape. Several models suggest that changes in cell wall components, such as cellulose or pectin, are involved in pavement cell formation, but views differ on the relationship between changes in the cell wall and turgor pressure. It was suggested that turgor pressure on the periclinal cell wall initiates the deformation of the cell wall [113,116] but also that turgor-independent heterogeneity of the pectin in the anticlinal wall is a major factor in this process [8,114].

The heterogeneity of cell wall properties along the periphery of pavement cells was identified using atomic force microscopy, and the corresponding asymmetric distribution of pectin components was visualized by electron microscopy [114]. These biochemical wall heterogeneities appear before wall bending, prompting the view that pectin-derived mechanical heterogeneity of the cell wall, rather than turgor pressure, might initiate a wavy cell contour. The recently suggested “expanding beam” model further supports this viewpoint [8]. Pectin HG forms nanofilaments in the anticlinal walls of pavement cells, and demethylation-mediated nanofilament inflation can guide cell shape in the absence of turgor-driven growth [8]. Understanding the association of nanofilaments with cellulose or other cell wall components, or the relationship of nanofilaments with the periclinal wall, remains a challenge for the future. A multifaceted discussion of controversial new results was provided in a recent review [117].

### 4.3. The Interplay between Auxin and Cell Wall Mechanics

The plant hormone auxin affects diverse aspects of growth and development [118,119,120], including cell expansion, which requires the loosening and remodeling of cell wall structures. The classic acid growth theory provides a conceptual framework for cell expansion in which auxin activates plasma membrane-localized H^+^-ATPases, leading to apoplast acidification. Reduced apoplastic pH leads to the activation of cell wall-loosening enzymes, which ultimately drive cellular expansion [121,122]. Auxins play an essential role in regulating apoplastic pH and the resulting cellular expansion in various tissues, including shoots and flowers [123,124,125].

Although the mechanisms underlying acid growth remain unclear, recent studies provided some clues to help unravel the relationship between auxin and cell wall mechanics. The degree of methylesterification of pectin, which is controlled by PME and PMEI activity, has a profound effect on the mechanical properties of the cell wall [35]. Since the enzymatic activities of PME and PMEI, as well as the formation of PME-PMEI complexes, are sensitive to pH [35,126,127,128], auxin-induced pH changes in the local cell wall environment can directly affect the degree of pectin methylesterification. Moreover, a recent study of apical hook formation revealed a spatial correlation between asymmetric auxin distribution, methylesterified pectin, and differential cell elongation [7]. Hook formation relies primarily on asymmetric cell elongation, in which high auxin levels on the inner side of the hypocotyl are associated with a reduction in cell elongation relative to the outer side, resulting in hypocotyl bending [129,130]. Spatial asymmetry was observed for pectin methylesterification levels, with high HG methylesterification levels correlating with slowly growing cells and the opposite trend on the outer, more rapidly growing side of the hypocotyl [7]. When the asymmetry of HG methylesterification is genetically perturbed by ectopically expressing *PMEI5*, cell elongation rates are reduced on the outer side of the hypocotyl, suggesting that differential cell elongation relies on HG methylesterification. HG methylesterification is enhanced in *yuc1D* mutants, which show enhanced auxin levels due to the increased expression of *YUCCA1*, encoding a rate-limiting enzyme in the indole-3-pyruvate pathway of auxin biosynthesis. This finding points to a positive association between auxin and HG methylesterification [7], which may be due to auxin-derived pH changes in the cell wall, although this was not directly shown.

The degree of methylesterification of pectin induced by auxin seems to differ depending on the cell type. During hook development, high auxin levels on the inner side of the hypocotyl mediate high methylesterification to limit growth [7], whereas during organ initiation, auxins favor demethylesterification of pectin to promote tissue softening [86]. These differential effects of auxins on HG methylesterification may reflect the diverse roles of auxins in organ initiation and differential growth, while some may be attributed to the cell-type specificity of PME and PMEI. PME and PMEI belong to large families whose members vary widely in terms of characteristics such as optimal pH, substrate specificity, and cell-type expression patterns, and whose specific functions are not well understood. The same pH change can lead to different outcomes of pectin methylesterification depending on which isoforms are predominantly expressed and the expression ratios of PME and PMEI.

Diverse, concentration-dependent roles of auxin in apoplastic pH homeostasis were also reported in roots, where auxin signaling is required for apoplast acidification, a process that is important for root cell expansion. High auxin levels transiently induce alkalinization of the root apoplast [101]. Asymmetric apoplast alkalinization is observed inside gravi-stimulated roots, where auxin levels are high, pointing to the biological relevance of high auxin concentrations in apoplast alkalization. Whether this involves altered plasma membrane H^+^-ATPase activity, pectin modifications, and/or molecular mechanisms shared with hook bending, are questions to be addressed in the future.

Given the relationship between auxin and the cell wall, many studies suggest a flow of auxin-cell wall signaling, but recent studies suggest the possibility of a feedback in which cell wall modifications affect auxin signaling. In xyloglucan-deficient *xxt1 xxt2* mutants, the auxin response maxima on the inner side of the hook are severely attenuated [131]. *xxt1 xxt2* mutants lacking detectable xyloglucan also show defects in the production and patterning of cellulose [132], which influence polar auxin transport by affecting PIN1 polarity [133]. Therefore, the defect in auxin distribution in *xxt1 xxt2* mutants could be due to an effect on the localization of auxin transporters. However, changes in the transcriptional regulation of specific auxin transporters are observed in *xxt1 xxt2* mutants [131]. The altered transcript levels of auxin transporters are suppressed in *xxt1 xxt2 arf2* mutants, suggesting that AUXIN RESPONSIVE FACTOR 2 (ARF2) mediates the interplay between the cell wall and auxin. It remains unclear as to how xyloglucan affects the transcriptional regulation of polar auxin transporters. The *xxt1 xxt2* mutants are defective not only in xyloglucan but also in cellulose and pectin [132,134], and it is difficult to determine which cell wall component is responsible for the transcriptional changes. Moreover, the transcriptional regulation may result from the recognition of changes in the mechanical integrity of the cell wall rather than changes in certain cell wall components such as xyloglucans or pectin. Finding answers to these questions could provide clues about the link between retrograde signals and hormones that reflect extracellular changes.

## 5. The Roles of Pectin in Cell–Cell Communication

In animals, cell–cell interactions frequently occur via direct contact between proteins located in the plasma membranes of neighboring cells. By contrast, plant cells are surrounded by cell walls, and direct contact between proteins located in adjacent cells is not possible. As a result, plant cells sense changes in surrounding cells differently. The propagation of microRNAs or proteins through plasmodesmata [135,136], and the recognition of diffusive factors such as small peptides by receptor-like kinases [137,138,139,140,141], have been proposed as major mechanisms of cell–cell communication in plants.

Recent studies of male–female interactions in plants showed that the cell wall, which blocks direct protein interactions, can mediate the recognition of surrounding cells [6]. FER receptor kinases play key roles in ensuring reproductive success, such as regulating the transfer of sperm to the female gametophyte inside the ovule or blocking female gametophyte penetration by multiple pollen tubes [142,143,144]. Duan et al. [6] explored the mechanisms underlying FER-mediated inhibition of polyspermy, revealing that nitrosylation of the precursor and mature forms of the chemoattractant LURE block its secretion and receptor interactions, respectively. Pectin is involved in the signaling link between FER and nitric oxide (NO). High levels of de-esterified pectins are present at the filiform apparatus, and genetic changes that reduce the level of de-esterified pectin increase the incidence of polyspermy. These findings, together with the observation that the production of de-esterified pectin is FER-dependent, suggest that de-esterified pectin in the filiform apparatus mediates the action of FER to prevent the entry of multiple pollen tubes into the female gametophyte [6].

Pectin also appears to participate in the accumulation of NO in the filiform apparatus, which is triggered by the arrival of pollen tubes at ovules. This NO accumulation can be mimicked in unpollinated pistils by applying pollinated pistillate exudate enriched in de-esterified pectin or commercially obtained de-esterified pectin to the ovules [6]. Treatment with structural components of pectin also stimulates NO accumulation in leaf discs [145] and in wild-type (but not *fer-4*) roots [6]. These observations suggest that the signaling linkage between de-esterified pectin, FER, and increased NO levels might explain other cell-cell communication processes as well.

The importance of cell-cell communication is also exemplified by the process of LR development. Since LR development initiates from a subset of pericycle cells located deep within the primary root [146], LR growth and emergence from the root surface require the cooperation of the three overlying cell layers (the endoderm, cortex, and epidermis) [147,148,149]. Communication between pericycle and endoderm cells occurs prior to the first cell division of the LR founder cells [149,150]. The transverse area of pericycle cells destined to form LRs increases before and during the first asymmetric cell division, while the overlying endoderm is slightly contracted or deformed. The increase in pericycle cell width is absent in *CASP1pro::shy2-2* plants, suggesting that these coordinated responses are dependent on endodermal auxin signaling at the earliest stages of LR development [149]. The detailed mechanism of the mechanical communication between the pericycle and the endodermis has not been elucidated, but since changes in pectin have effects as early as the initial stage of LR development [9], it is possible that pectin mediates communication between the two cell types.

During LR development, cell wall remodeling in the overlying cell layers is required to allow for the passage of the expanding LRP. The expression of genes encoding many cell wall-remodeling enzymes, including pectin-modifying enzymes, is regulated in an auxin-dependent manner [151]. Auxin-induced cell wall remodeling during LR development is mediated by HAESA (HAE), HAESA-LIKE2 (HSL2), and their ligand INFLORESCENCE DEFICIENT IN ABSCISSION (IDA), in a process that is similar to flower organ abscission [148,152]. The auxin-induced expression of *IDA* is detected by the HAE/HSL2 receptor and activates the downstream MITOGEN-ACTIVATED PROTEIN KINASE3 (MPK3) and MPK6 cascades. These signaling cascades increase the expression of cell wall remodeling genes, resulting in pectin degradation and cell wall separation, which are required for LR emergence. Mutants with suppressed expression of a cell wall enzyme show changes in LR emergence, thus confirming that cell wall remodeling in the overlying cells is required for this process [150,153,154]. Whether the phenotype of cell wall mutants is due to a passive change in the mechanical properties of the overlying cell layers or an active influence on the LRP remains an interesting issue.

## 6. The Roles of Pectin in Plant–Environment Interactions

### 6.1. Abiotic Stress Responses

**Figure 2:**
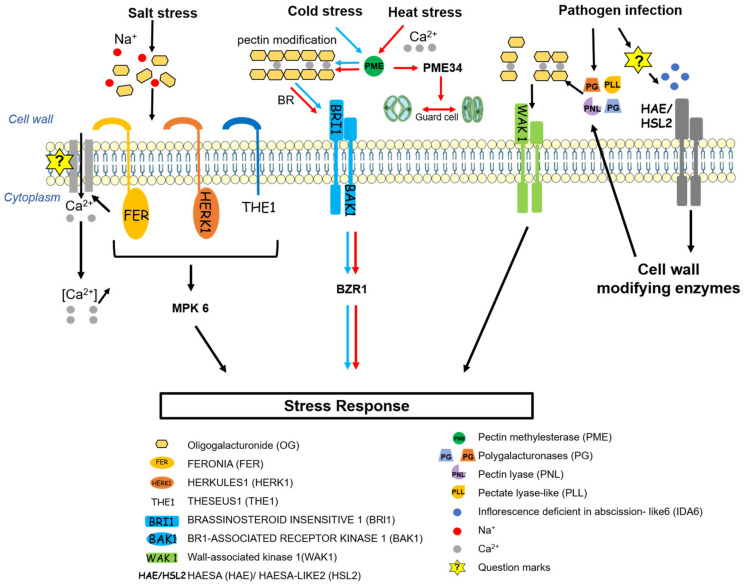
The roles of pectin in signaling processes during interactions with the environment. Salt stress affects the cell wall because sodium ions disrupt pectin crosslinking. The cell wall sensor FERONIA (FER), in combination with HERKULES1 (HERK1) and THESEUS1 (THE1), senses salinity through its pectin-binding extracellular domain, leading to an increase in cytosolic [Ca^2+^] and mitogen-activated protein kinase 6 (MPK6) function, which are required for salt-induced gene expression. Cold stress induces the expression of pectin methylesterase (PME) and modulates the rigidity of the cell wall via brassinosteroid (BR) signaling. BR molecules are perceived by BRI1, triggering the formation of BRI1-BAK1, which initiates an intracellular phosphorylation relay cascade and activates BZR1. Heat stress in combination with Ca^2+^ increases the rate of demethylesterification via PME activity. PME34 is involved in regulating stomatal aperture during heat stress. During pathogen infection, pectin is degraded to produce oligogalacturonides (OGs), which are detected by wall-associated kinase 1 (WAK1), leading to a strong immune response. The pathogen secretes polygalacturonase (PG), pectin lyase (PNL) and pectate lyase-like (PLL) to degrade pectin and attempt infection. It also regulates *IDL6* expression and the downstream HAE/HSL2 pathways to produce pectin-degrading enzymes from the host. Question marks represent an unknown receptor, MPK, or transcription factor.

The plant cell wall, which constitutes the outermost protective layer of the organism, plays an important role in plant responses to a variety of environmental conditions. In particular, pectin is involved in plant responses to abiotic stresses such as saline stress, osmotic stress, and temperature changes (Figure 2). Salinity simultaneously induces ionic and osmotic stress, triggering subsequent signaling responses that are detrimental to plant growth [155]. Despite much effort, it is still not known how plants sense salt. Plant cell wall modification might be a factor that regulates the salt stress response [156]. Salinity affects the mechanical properties of the cell wall in the root tip region, likely because sodium ions directly disrupt pectin crosslinking within the wall. It is essential for plants to rapidly recognize such damage and regulate the downstream cellular events required to prevent the loss of integrity. FER is responsible for sensing salinity-induced cell wall defects via its pectin-binding extracellular domain, which leads to a rapid, cell-autonomous increase in cytosolic [Ca^2+^]. This increase, in turn, regulates downstream cellular events that are required to prevent cell bursting under high salinity [156]. In addition to FER, HERKULES1 (HERK1) and THESEUS1 (THE1) act as sensors of pectin-associated damage to the cell wall, and MPK6 is partially implicated in eliciting downstream responses [157].

Freezing is another abiotic stress that damages plants. The plasma membrane of the cell is the primary site of damage by freezing in plants, and during the freeze–thaw cycle, it ruptures, leaking the contents of the cytoplasm [158]. Since the concentration of solute in the extracellular fluid is lower than the concentration of the intracellular fluid, ice formation generally begins in the intercellular space, and the water potential outside the cell falls, causing unfrozen water to move down the chemical potential gradient and eventually leading to disruption of the plasma membrane and cell death [159,160]. Therefore, the increasing of wall thickness and strength through the utilization of cell wall components including pectin can be considered a key strategy to withstand freezing [161,162,163]. Transcriptome and proteome analyses indicate that cell wall remodeling is particularly important to increase freezing tolerance during cold acclimation [164,165,166]. The abundance of several PMEs and PMEIs is significantly affected during sub-zero acclimation [164], and brassinosteroid (BR) signaling plays a role in regulating PME activity [162]. Reducing PME activity through the overexpression of *PMEI*, or reducing RG-II dimerization via mutations in the *MUR1* gene, results in lower freezing tolerance compared to wild-type plants [167,168]. These findings suggest that cell wall remodeling, including crosslinking of HG and RG-II, plays an important role in freezing tolerance.

Strategies that modulate the mechanical properties of the cell wall via pectin modifications may be important not only for cold acclimation [162] but also for the heat response [169,170,171]. Heat stress is defined as a rise in temperature that induces irreversible damage to plant growth and development. Plants exposed to sublethal temperatures develop thermotolerance that allows them to survive subsequent exposure to normally lethal high temperatures; the accumulation of heat shock proteins is part of this process. Cell wall remodeling is also an integral part of the heat response network. The rate of demethylesterification via PME activity increases substantially with increasing temperature [172]. Together with Ca^2+^ mobilization from apoplastic sources, the enhanced activity of PME results in cell wall remodeling, a process that is important for heat tolerance [171]. PME34 is involved in the regulation of cell wall flexibility in guard cells and contributes to heat tolerance by regulating the rate of transpiration in response to heat stress [170]. This study sheds light on how the regulation of cell wall dynamics by PME can contribute to specific physiological functions in response to environmental stress.

BR signaling is required for plant growth [173,174], and BR-treated plants show increased resistance to various abiotic stresses [175]. Several studies have attempted to elucidate the role of BR in plant growth control by linking it with pectin-dependent cell wall homeostasis [93,176,177]. Genetic or pharmacological interference with PME activity results in dramatic changes in plant growth behavior and activation of the BR signaling pathway [176], in which RECEPTOR-LIKE PROTEIN 44 (RLP44) serves as a feedback signaling module [177]. RLP44 promotes BR signaling by interacting with the BR receptor BRI1 and its co-receptor BRI1-ASSOCIATED KINASE 1 (BAK1) [177,178]. A recent study investigating the effects of cellular phenotype on BRI1-dependent macroscopic growth defects such as reduced root length and root waving revealed a role involving the cell wall and BR signaling in cell wall orientation [93]. It remains unclear as to how *PMEI5* expression in the cortex leads to oblique cell divisions in both epidermal and cortical cells, how the cell wall maintains the orientation of cell division, and how the cellular effects of BR are linked to organ-level responses. The answers to these questions might help to explain how cell wall homeostasis and coordinated growth are maintained.

### 6.2. Plant Defense Mechanisms

The cell wall provides the first line of defense in plants, playing a role in disease resistance specificity and fitness [179]. Biochemical profiling using synchrotron-based spectroscopy visualizes the changes in the biochemical spectrum of cell wall components in relation to host defense responses [180,181]. Spectroscopic results show increased peak intensity in cellulose, hemicellulose, pectin and glucan, suggesting that these structural components have a role in the response to pathogen infection.

Microorganisms have developed a variety of strategies to break down this physical barrier (Figure 2). Microorganisms secrete a series of pectin enzymes to directly break down de-esterified HG and attempt infection [182]; they also hijack the host signaling pathway that induces cell wall remodeling [183]. IDA and HAE/HSL2-mediated signaling pathways are involved in controlling floral organ abscission and LR emergence, promoting the expression of genes encoding various cell wall-digesting enzymes, particularly PG [148,152,184,185]. The IDA family member *IDL6* is upregulated by infection with the pathogen *Pst* DC3000, activating the downstream HAE/HSL2 pathways, which in turn promote pectin digestion in leaves and increase sensitivity to *Pst* DC3000 [183]. Although the molecular mechanism inducing the increase in *IDL6* expression remains largely unknown, these results suggest that *Pst* DC3000 can enhance infection by hijacking the IDL6-HAE/HSL2 signaling pathway. An effector protein that binds to pectin and interferes with pectin crosslinking was also discovered [186]. During the infection of tomato with the fungal pathogen *Pseudocercospora fuligena*, the fungal effector PfAvr4-2 is secreted, interacting with de-esterified pectin and disrupting Ca^2+^-mediated crosslinking at the cell-cell junction to loosen the plant cell wall structure and enhance the activity of pathogen-derived pectinases [186].

Pectic fragments from damaged cell walls are released via the activation of these pectic enzymes and detected by the host as danger signals, called damage-associated molecular patterns (DAMPs), which result in the activation of the innate immune response. Oligogalacturonides (OGs), the breakage products of HG, are a major source of cell wall-derived DAMPs [187,188]. A recent biochemical analysis with a high-resolution separation method revealed the global OG composition of plant samples during *Arabidopsis thaliana* infection with the fungus *Botrytis cinerea* [189]. Unmethylesterified OGs, which were considered important DAMPs for necrotrophic pathogens and frequently used in the study of defense responses [187,190,191,192], were absent in *B. cinerea*-infected citrus pectins or *A. thaliana* leaves. Instead, most of the OGs were acetyl- and methylesterified, and 80% were produced by fungal PNLs, not PGs [189]. This study also showed that the OG profiles generated by *B. cinerea* grown on citrus pectins and on *A. thaliana* leaf cell walls were significantly different, suggesting that the complexity of OGs reflects the arms race of plant–fungus interactions in vivo.

OGs are produced not only by pathogens but also by plant-derived enzymes. Plants produce PG inhibitory protein (PGIP) against fungal PGs, slowing PG activity and preventing the complete hydrolysis of HG. This process increases the accumulation of OGs, which can act as endogenous DAMPs [187]. On the other hand, a pathway by which OGs are generated during cell wall remodeling was reported when recognizing engineered structural perturbations of the cell wall [193]. Genetic modification of plant cell wall polymers was attempted in order to improve lignocellulosic biomass, and these attempts often led to ectopic activation of defense responses and a decrease in biomass yield [193,194,195]. The reduction in lignin content via mutations of monolignol biosynthetic enzymes induces an increase in *PG* expression, including that of *ARABIDOPSIS DEHISCENCE ZONE POLYGALACTURONASE 1* (*ADPG1*), thereby increasing the release of size- and charge-heterogeneous pectic OG elicitors to induce pathogenesis-related (PR) gene expression [193]. These results highlight the importance of pectin for defense signaling as well as in cell wall remodeling.

Arabidopsis wall-associated kinase 1 (WAK1) acts as a receptor for OGs, thereby playing a role in innate immunity [196,197]. A recent study further elucidated the mechanism of action related to the binding of WAK1 to pectin in the desiccation-tolerant resurrection plant *Craterostigma plantagineum* [198]. CpWAK-pectin binding requires the formation of CpWAK multimers and is regulated by *C. plantagineum* glycine-rich protein1 (CpGRP1) and apoplastic pH. These observations indicate that CpGRP1, apoplastic pH and Ca^2+^, and the formation of CpWAK multimers are potential factors for discriminating and integrating cell wall signals generated by diverse stimuli.

Although OGs help to develop improved resistance to several pathogens, the prolonged release of OGs is deleterious and causes reduced plant growth, suggesting the importance of OG homeostasis as a regulator of the growth–defense trade-off. Recently, an enzyme that oxidizes OGs was identified and named OG OXIDASE 1 (OGOX1) [199]. OGOX1 is a sulfite-sensitive H_2_O_2_-generating enzyme and belongs to a large family of Berberine Bridge Enzyme-like (BBE-like, 28 members) enzymes, of which at least four are OG oxidases (OGOX1–4) [200]. Oxidized OGs are more resistant to hydrolysis by fungal PGs but have a reduced ability to activate immune responses. Cellodextrins (CDs), cellulose breakdown products that act as DAMPs, are also regulated in a similar way [201]. CDs are oxidized by another Arabidopsis BBE-like protein (CD3-6) and exhibit reduced elicitor activity after oxidation. These results suggest that inactivation of OGs and CDs may be a general strategy of plants for controlling the homeostasis of DAMPs and the growth–defense trade-off.

## 7. Conclusions

Evolution began with a single-celled organism surrounded by a cell wall. One pathway in the evolution of multicellular organisms involved shedding the hard coats of cells, which made cell migration and intercellular protein–protein interactions possible, giving rise to animals. Another pathway, in which thick cell walls were maintained, led to the evolution of plants. It remains largely unknown how the cellular specification and interactions that are essential for the construction of multicellular organisms arise and are maintained in plants, along with the presence of cell walls that impose great restrictions on cell-cell interactions and cellular movement. Recent studies examining the diversity, dynamics, and variability of cell wall components, especially pectin, and their effects on intercellular pathways provided clues about the fundamental features of plant multicellularity. These studies propose that plant cells do not exist as islands surrounded by insulating cell walls but as a continuum of cell wall-plasma membrane-cytoplasm. This new paradigm suggests that the cell wall is directly involved in various cellular processes as an active participant rather than a passive executor of commands. Further studies are needed to understand the underlying mechanisms in detail, such as how cell wall diversity and plasticity are specifically regulated, how protein dynamics and specificity are regulated in the extracellular space, and which components are involved in securing extracellular and intracellular connectivity. These are crucial questions about the roles and regulatory mechanisms of the cell wall, and answering them will bring us closer to understanding the key features of plant multicellularity that evolved along with the cell wall.

## Data Availability

Not applicable.
